# Activity tracker-based intervention to increase physical activity in patients with type 2 diabetes and healthy individuals: study protocol for a randomized controlled trial

**DOI:** 10.1186/s13063-022-06550-z

**Published:** 2022-07-30

**Authors:** M. Mähs, J. S. Pithan, I. Bergmann, L. Gabrys, J. Graf, A. Hölzemann, K. Van Laerhoven, S. Otto-Hagemann, M. L. Popescu, L. Schwermann, B. Wenz, I. Pahmeier, A. Teti

**Affiliations:** 1 Institute of Gerontology, Vechta University, Vechta, Germany; 2 Sport Science, Vechta University, Vechta, Germany; 3 University of Applied Sciences for Sport and Management Potsdam, Potsdam, Germany; 4 Research group Ubiquitous Computing, University of Siegen, Siegen, Germany; 5 Diabetologische Schwerpunktpraxis Dr. Silke Otto-Hagemann (diabetes center) Vechta, Vechta, Germany

**Keywords:** Randomized controlled trial, Physical activity, Diabetes, Activity tracker, Wearables, Motivation and volition

## Abstract

**Background:**

One relevant strategy to prevent the onset and progression of type 2 diabetes mellitus (T2DM) focuses on increasing physical activity. The use of activity trackers by patients could enable objective measurement of their regular physical activity in daily life and promote physical activity through the use of a tracker-based intervention. This trial aims to answer three research questions: (1) Is the use of activity trackers suitable for longitudinal assessment of physical activity in everyday life? (2) Does the use of a tracker-based intervention lead to sustainable improvements in the physical activity of healthy individuals and in people with T2DM? (3) Does the accompanying digital motivational intervention lead to sustainable improvements in physical activity for participants using the tracker-based device?

**Methods:**

The planned study is a randomized controlled trial focused on 1642 participants with and without T2DM for 9 months with regard to their physical activity behavior. Subjects allocated to an intervention group will wear an activity tracker. Half of the subjects in the intervention group will also receive an additional digital motivational intervention. Subjects allocated to the control group will not receive any intervention. The primary outcome is the amount of moderate and vigorous physical activity in minutes and the number of steps per week measured continuously with the activity tracker and assessed by questionnaires at four time points. Secondary endpoints are medical parameters measured at the same four time points. The collected data will be analyzed using inferential statistics and explorative data-mining techniques.

**Discussion:**

The trial uses an interdisciplinary approach with a team including sports psychologists, sports scientists, health scientists, health care professionals, physicians, and computer scientists. It also involves the processing and analysis of large amounts of data collected with activity trackers. These factors represent particular strengths as well as challenges in the study.

**Trial Registration:**

The trial is registered at the World Health Organization International Clinical Trials Registry Platform via the German Clinical Studies Trial Register (DRKS), DRKS00027064. Registered on 11 November 2021.

## Administrative information

Note: the numbers in curly brackets in this protocol refer to SPIRIT checklist item numbers. The order of the items has been modified to group similar items (see http://www.equator-network.org/reporting-guidelines/spirit-2013-statement-defining-standard-protocol-items-for-clinical-trials/).

**Table:** 

Title {1}	Activity tracker-based intervention to increase physical activity in patients with type 2 diabetes and healthy individuals: study protocol for a randomized controlled trial
Trial registration {2a and 2b}.	The trial “Activity tracker-based intervention to increase physical activity in patients with type 2 diabetes and healthy individuals” is registered at the World Health Organization International Clinical Trials Registry Platform via the German Clinical Studies Trial Register (DRKS), DRKS00027064. Registered on 11 November 2021; URL: https://www.drks.de/drks_web/navigate.do?navigationId=trial.HTML&TRIAL_ID=DRKS00027064.
Protocol version {3}	Protocol version 7 14 June 2022
Funding {4}	This trial is part of the project ActiVAtE_Prevention – “Activity Tracking Data to Understand Volition, Attrition and Engagement towards Healthy Behaviors in Diabetic Patients and Controls” that is funded by the German Ministry for Science and Culture of Lower Saxony in the program “Niedersächsisches Vorab” (funding code VW-ZN-3426).
Author details {5a}	^1^Vechta University, Institute of Gerontology, Germany ^2^Vechta University, Sport Science, Germany ^3^University of Applied Sciences for Sport and Management Potsdam, Germany ^4^University of Siegen, Research group Ubiquitous Computing, Germany ^5^Diabetologische Schwerpunktpraxis Dr. Silke Otto-Hagemann (diabetes center) Vechta, Germany
Name and contact information for the trial sponsor {5b}	Overall responsibility for the initiation and management of the trial: Vechta University, Germany Contact name: Mr. Andrea Teti Address: Driverstraße 23, 49377 Vechta Telephone: +49 4441 15 791 Email: andrea.teti@uni-vechta.de
Role of sponsor {5c}	The funding body had no role in the design of this study. It will not have any role during its execution, analyses, interpretation of the data, or on the decision to submit results.

## Introduction

### Background and rationale {6a}

Type 2 diabetes mellitus (T2DM), which results in elevated blood sugar levels (hyperglycemia) due to insulin resistance, is one of the most common chronic diseases among adults [1]. Hyperglycemia and diabetes mellitus are associated with micro- and macrovascular disorders such as coronary heart disease, stroke, renal and eye disease, as well as an increased risk of developing certain types of cancer and depressive symptoms [2–4]. Genetic predisposition, age, and lifestyle factors, such as a sedentary lifestyle, an unhealthy diet, and smoking, are associated with a higher risk of developing T2DM [5–7]. Therefore, one relevant strategy to prevent the onset of the disease in healthy individuals and to slow or halt disease progression and the development of secondary diseases in people with T2DM focuses on increasing physical activity [8–13].

Studies suggest that physical activity can reduce fasting blood glucose (FBG) and average blood sugar concentrations (measured by glycated hemoglobin (HbA_1c_)) in healthy individuals [14–16]. These reductions could lower the risk of hyperglycemia and thus the risk of developing T2DM [15]. According to the results of a meta-analysis, compliance with the World Health Organization (WHO) recommendation to be physically active at a moderate level of intensity for at least 150 min per week would result on average in an FGB reduction of 4.13 mg/dL and 0.21% lower HbA_1c_ in healthy individuals [14]. In addition, the results of Cavero-Redondo et al. found that engaging in more than 150 min of physical activity per week could lead to a greater reduction in HbA_1c_ levels compared with having lower levels of physical activity [16].

Research also suggests that physical activity can reduce FBG and HbA_1c_ levels in people with T2DM or pre-diabetes [11, 14, 17, 18]. Based on the previously mentioned meta-analysis, following the WHO recommendation for moderate physical activity for at least 150 min a week would result on average in an FBG reduction of 7.07 mg/dL and 0.24% lower HbA_1c_ in people with T2DM or pre-diabetes [14]. In addition, large-scale cohort studies have shown that physical activity significantly reduces the risk of mortality [11].

Physical activity can be increased not only through structured programs, such as aerobic or resistance training, but also through unstructured leisure time physical activity [13]. Valid, representative, and objectively measured data are needed to understand the complex factors affecting physical activity in individuals’ daily routines [19]. Activity trackers are promising tools for objectively collecting physical activity data and increasing physical activity in different target populations [20–25]. They are built into consumer electronics (so-called wearables), such as smartphones, smartwatches, and fitness bracelets, and regularly collect physical activity data during everyday life; the processed data are then reported to the end-user [26].

Several studies from different countries show promising results regarding the use of activity trackers:

Baskerville et al. conducted a meta-analysis based on 12 randomized controlled trials or controlled trials ((R)CTs) using pedometers and accelerometers (in combination with behavioral interventions in some studies) that suggested that these interventions could lead to a statistical significant increase in physical activity of about 1 hour per week and a statistically significant reduction in HbA_1c_ levels of about 0.1 mmol/mol in T2DM patients [27]. However, no statistically significant effects were found regarding changes in blood pressure, body mass index (BMI), and lipid values [27].

The meta-analyses conducted by Gal et al. based on 18 randomized controlled trials (RCTs) and by Brickwood et al. based on 12 (R)CTs, in which activity tracker-based consumer electronics in combination with behavioral interventions were used, indicate a statistically significant increase in steps (standardized mean difference (SMD): 0.67 and 0.23) as well as in moderate to vigorous physical activity per day (SMD: 0.49 and 0.28) [20, 28]. However, with the use of the wearable intervention alone, the study results of Brickwood et al. only suggested a statistically significant increase in steps (SMD: 0.20; mean difference (MD): 475 steps per day) but not in moderate to vigorous physical activity per day (SMD: 0.17; MD: 40 min per day) [20].

The effectiveness of activity tracker-based interventions in everyday life could be influenced by user behavior, user satisfaction, perceived effectiveness and usefulness of the technology, and technology acceptance in general [29, 30]. Furthermore, the validity and reliability of the data collected by activity trackers could be affected by technical problems associated with the device, changes made to the device (e.g., software updates), and incorrect use of the device by the person using it or by the study personnel [31]. A systematic review conducted by Bort-Roig et al. reported that the accuracy of physical activity data (steps per day) measured with smartphones ranged from 52 to 100% [21]. Regarding the accuracy of fitness trackers, a systematic review of Fitbit devices suggested an overall tendency to underestimate steps (MD: − 9%) [32].

In previous studies in which activity trackers were used to monitor and enhance physical activity, several limitations were mentioned, including small sample sizes, short study durations, lack of standardized analytical procedures, and further research needs, such as the use of tracker interventions in subjects with T2DM [20, 26–28, 33–36]. Therefore, a RCT with a longer study duration and larger sample size relative to former studies will be conducted to investigate the effectiveness of an activity tracker-based intervention alone and in combination with a behavioral intervention. Healthy individuals, individuals with pre-diabetes and individuals with T2DM will be included in the study to assess the benefits of the intervention regarding risk reduction for the development of T2DM onset as well as disease progression. The aim of this trial is to answer the following research questions: (1) Is the use of activity trackers suitable for longitudinal assessment of physical activity in everyday life? (2) Does the use of a tracker-based intervention lead to sustainable improvements in the physical activity of healthy individuals and in people with T2DM? (3) Does the accompanying digital motivational intervention lead to sustainable improvements in physical activity for participants using the tracker-based device?

### Objectives {7}

In this trial, it is hypothesized that the moderate and vigorous physical activity of the participants in the groups (no diabetes, diabetes and pre-diabetes) with activity tracker increases statistically significantly in terms of the target values at the 5% level of significance over time (at baseline (*t*_0_), after 3 months (*t*_1_), after 6 months (*t*_2_) and after 9 months follow-up (*t*_3_)). The measurements of moderate and vigorous physical activity with questionnaire are hypothesized to be statistically significantly different at the 5% level of significance from the measurements of physical activity with the activity tracker. In addition, an explorative hypothesis is tested: The moderate and vigorous physical activity in minutes per week measured with the activity tracker of participants in the different intervention groups (tracker-based intervention alone or tracker-based intervention combined with digital motivational intervention) increases differently over time.

### Trial design {8}

The monocentric, pragmatic RCT has a parallel group design with an allocation ratio of 1:1. Tests of equivalence will be performed to compare the intervention group wearing a smartwatch with an activity tracker in everyday life with the control group that does not wear the device. Furthermore, 50% of the participants in the intervention group will also receive a digital motivational intervention via a smartphone app. The planning and implementation of the study is orientated towards the Declaration of Helsinki [37].

## Methods: Participants, interventions, and outcomes

### Study setting {9}

The study is conducted in the rural region around Vechta, a town in the northwestern part of Germany. The data collection is done at a cooperating specialized diabetes center in Vechta while trackers are worn by participants during everyday life.

### Eligibility criteria {10}

#### Inclusion criteria:

All participants have to provide written informed consent before enrollment in the study. Having a smartphone and internet access is required for study participation to ensure data collected with the tracker-based device can be transmitted to the research institution.

#### Exclusion criteria:

For safety reasons, the general exclusion criteria are age < 18 years, legal guardianship, pregnancy, and an increased risk of falling. In addition, diabetes patients with the following comorbidities and contraindications are excluded [13]: peripheral neuropathies, diabetic foot syndrome, myocardial infarction or stroke diagnosed in the previous 4 weeks, insufficiently controlled high blood pressure (≥140/90 mm Hg), hypoglycemia perception disorders, psychiatric disorders, poorly controlled T2DM (≤ 3 weeks therapy), severe sensorimotor and/or autonomic neuropathy, and unstable proliferative retinopathy.

### Who will take informed consent? {26a}

All individuals interested in participating in the trial receive an information sheet with detailed descriptions of the procedures, objectives, possible harms as well as benefits, and contact people related to the trial. Trained study personnel are available to answer questions regarding the trial and for explaining the procedures in detail. In addition, there are separate information sheets for healthy individuals and individuals with T2DM to provide precise information regarding possible risks associated with participation in the trial. The study personnel will obtain written informed consent from all individuals willing to participate in the trial.

### Additional consent provisions for collection and use of participant data and biological specimens {26b}

Detailed information about data collection procedures and the use and protection of participant data are provided on the information sheet. No additional consent is obtained because no ancillary studies or studies involving biological specimens are planned.

## Interventions

### Explanation for the choice of comparators {6b}

Because there are no suitable sham or placebo interventions, participants in the control group do not receive any intervention. In the intervention group, participants wearing only the tracker-based device are compared with participants wearing the tracker-based device and receiving a digital behavioral intervention via a smartphone app. This procedure is chosen to analyze the sole effect of the tracker and to determine a possible additional effect of a digital behavioral intervention accompanying the tracker-based intervention. All participants in the trial are given a brochure with detailed information about the relevance of physical activity for their health and national physical activity recommendations for different age groups [38] to strengthen their participation.

### Intervention description {11a}

The intervention group receives an activity tracker-based open-source smartwatch that is freely programmable [39–41]. Hence, firmware and applications specifically programmed for this study are used in order to comply with data protection and data security requirements. All device sensors that are not needed for the study are switched off for the entire study period.

The device will be tested in a separate study before the start of the main study to assess its accuracy and reliability. This assessment will involve a two-step process: (1) use under standardized laboratory conditions on a treadmill and (2) use under real-life conditions for 1–2 consecutive days. Participants (*n*=30) will wear the device and two reference devices (GT9X) on their wrist and hip. The Actigraph GT9X accelerometer is widely used in scientific studies to measure physical activity and shows good validity and reliability [42, 43]. The goal of this validation study is to define accurate cut-points and algorithms to detect physical activity levels (light, moderate, and vigorous) and steps. Further pre-studies with university students (*n*=25) wearing the tracker for 1 week will be conducted to assess the functioning as well as usability of the device and app, the practicability of the study design, and the questionnaires.

The smartwatch is linked to a smartphone app that is developed specifically for this study to protect the privacy of the participants and that transfers activity tracker data securely to the university server. In addition, the app is scientifically based and therefore provides the participants with assistance via behavior change techniques (BCT) that have been proven to be effective in changing behavior, specifically through self-monitoring, goal setting, and feedback [44–46]. Therefore, the app displays the steps and low, moderate, and vigorous physical activity in minutes per day (BCT “self-monitoring”), which are measured by the tracker-based device for each participant wearing the smartwatch. In addition, 50% of the participants wearing the tracker can set goals for themselves (BCT “goal-setting”) in terms of the physical activity levels they want to reach with the help of the app.

All participants in the intervention group receive a standardized introduction to the use of the device and the smartphone app as well as an instruction manual. In addition, qualified personnel will be trained to answer questions about the use of the device and the app and to offer support during weekly consultation hours until the end of the study in the case of technical problems.

### Criteria for discontinuing or modifying allocated interventions {11b}

Study participation does not affect medical treatment of diabetes patients, and the assignment of the patients to the intervention or control group does not have any effect on medical treatment in the diabetes center. Owing to regular visits to the center in line with the routine treatment, possible harms associated with the study can be monitored on a regular basis. Decisions about study drop-out due to medical reasons are based on the involved medical professionals’ judgment. The risks associated with monitoring and changing physical activity behavior are regarded as minor. The risk of hypoglycemia due to exercise is mentioned in the introduction to the device, and strategies are presented to reduce the risk. Patients who are at particularly high risk for hypoglycemia are excluded from participating in this study.

### Strategies to improve adherence to interventions {11c}

Previous study results have indicated that interventions involving tracker-based consumer electronics may contribute to adherence to behavioral recommendations [21, 47]. Nevertheless, adherence to wearing the tracker-based device may be low or may decline during the study. At baseline, the participants in the control group are instructed to wear the device during the day and to charge its battery overnight. In addition, the importance of wearing the device regularly is emphasized. Because the tracker-based intervention is applied and its effectiveness is tested in everyday life, no further measures to control adherence are taken.

### Relevant concomitant care permitted or prohibited during the trial {11d}

Because it is not possible to prevent participants from taking part in classes or other interventions that promote a healthy lifestyle, the participation in such interventions is queried at each measurement point and the results of the statistical analyses will be adjusted for such effects if necessary.

### Provisions for post-trial care {30}

Because the conduct of this study does not affect the medical treatment of the study participants and the risks associated with trial participation are negligible, no ancillary or post-trial care, irrespective of the routine care of the diabetes patients, are provided.

### Outcomes {12}

The primary outcome is a change from baseline of moderate and vigorous physical activity in minutes or steps per week. Therefore, moderate and vigorous physical activity levels are aggregated and measured by one continuous variable. Physical activity is measured continuously with the tracker-based device, and algorithms are implemented to calculate amounts of different intensities [41]. Intensity levels are defined in accordance to intensity classification of Freedson et al. [48].

In addition, physical activity is assessed with questionnaires at four time points (*t*_0_, *t*_1_, *t*_2_, and *t*_3_).

As the analysis of the patient-relevant outcomes morbidity, mortality, and comorbidity of diabetes patients requires long-term studies, which are not feasible in this study due to limited project resources, the clinical outcomes HbA_1c_, body weight, and blood pressure are used as relevant surrogate parameters for these outcomes [13]. Therefore, secondary endpoints are medical parameters (HbA_1c_, TIR (time in range), BMI, and blood pressure). As covariates, psychosocial parameters (generic or diabetes-specific: health-related life satisfaction, self-efficacy, motives, volition, stages of behavioral change, personality, intention, and self-concordance), socio-demographic variables (age, gender, family background, family status, and socio-economic status), physical and mental health, comorbidities, alcohol as well as tobacco consumption, nutritional awareness, usage behavior and user satisfaction (with regard to the activity tracker and smartphone app), diabetes disease and treatment (e.g., medication), participation in external interventions, and severity of the disease are considered. The medical parameters of the healthy individuals are measured at four time points and those of the diabetes patients are collected via routine visits at the cooperating diabetes center. The data about covariates are captured with questionnaires at the same four time points. The items of the questionnaires originate from validated instruments.

In general, changes in the arithmetic means and variances of these mentioned outcomes from baseline are analyzed and compared between the intervention and control groups as well as between the four time points.

### Participant timeline {13}

**Fig. 1:**
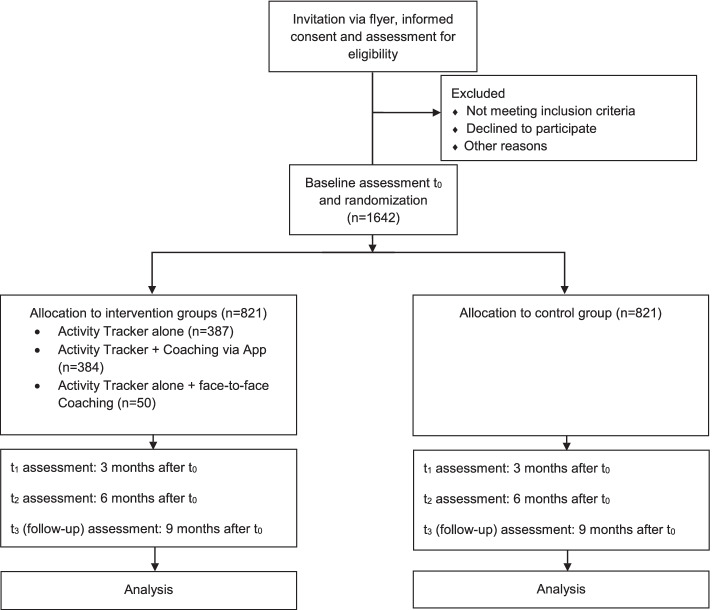
Graphical representation of the study design according to Consolidated Standards of Reporting Trials (CONSORT, [49, 50])

Participants who give their informed consent and fulfill the inclusion criteria are assessed at four timepoints (see Table 1 and Fig. 1). At baseline, a questionnaire with socio-demographic questions [51] is administered, and at all four time points, a questionnaire with health-related questions [52], questions regarding physical activity [53, 54], a psychosocial questionnaire [55–64], and questions regarding the use of tracker-based devices and apps outside this study is administered. In addition, the diabetes parameters are measured in a standardized way by qualified study personnel at all four time points. Questions regarding the usability of and satisfaction with the app and the tracker-based device [65] are asked after 6 months and regarding the follow-up use of the device after 9 months.

**Table 1:**
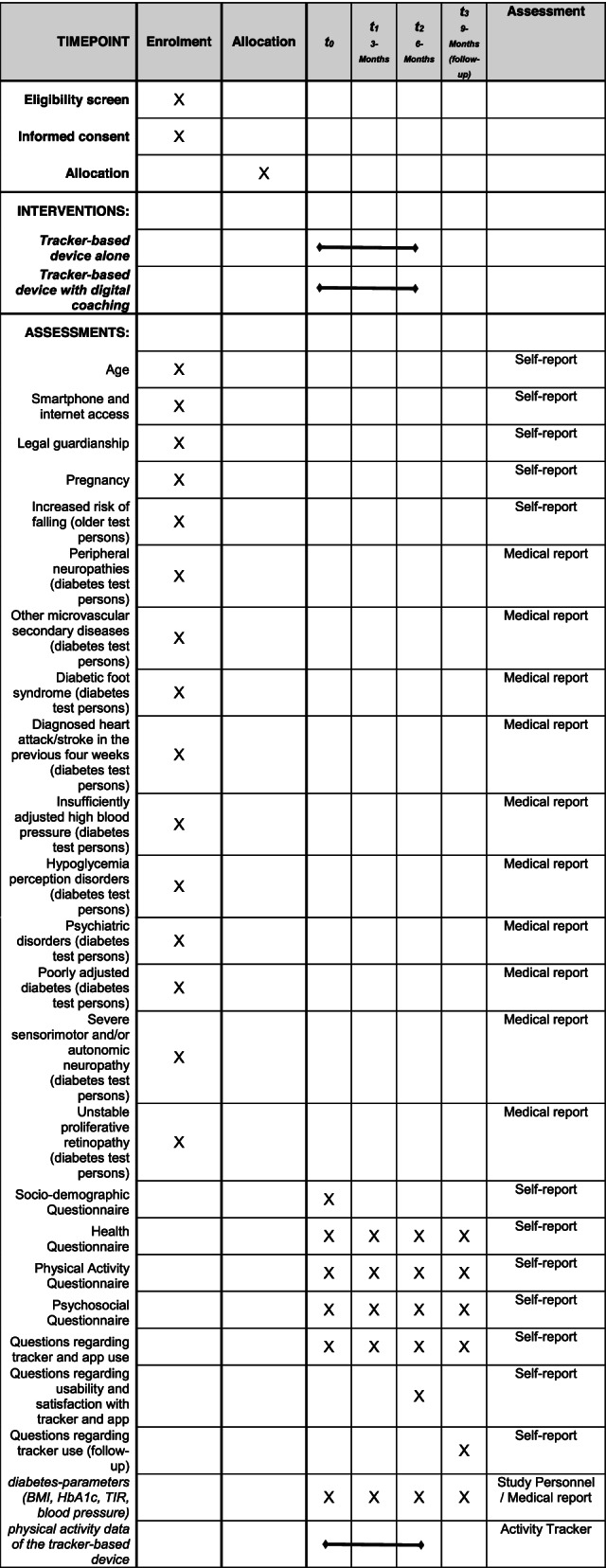
Participant timeline

At baseline, participants are randomly assigned to either the intervention or the control group. For participants in the intervention group wearing the tracker-based device, physical activity is measured continuously with the device for 6 months. For the remaining 3 months, participants can decide on their own if they want to continue wearing the tracker until t_3_. At t_3_ they have to return the tracker to the study personnel.

### Sample size {14}

The sample size was calculated according to the primary hypothesis. The correlation between the results of the different measurement times is estimated to be 0.33 based on similar studies [66]. Regarding the effect size, the results of similar studies show that a medium effect can be assumed, which is why the effect size is estimated at *d*=0.5 and *f*^2^=0.15 [27, 28, 33, 66]. A dropout rate of 15% is derived from one of these studies [66]. For a power (1-β) of 0.8 with a significance level of *α*=0.05, assuming an effect size of *f*^2^=0.15, an adjustment with design effect, and a drop-out rate of 15%, a sample size of at least 1642 participants is necessary. With the help of the results of the pre-studies, a more precise calculation of the sample size is possible and the sample size can be adjusted if necessary.

### Recruitment {15}

The enrollment period will extend over 12 months. The diabetes patients are recruited at the cooperating diabetes center and the healthy individuals at a cooperating company located in the same region. Brochures with information about the study and contact details are disseminated, and individuals in the diabetes center are personally and directly invited to participate in the study by the health practice staff.

## Assignment of interventions: allocation

### Sequence generation {16a}

In this RCT, subjects are assigned to the intervention and control group in a block-randomized manner stratified by disease status (no disease, pre-diabetes, diabetes) with an allocation ratio of 1:1. The block randomization is chosen to generate undistorted and approximately equal comparison groups. The block randomization itself is carried out on the basis of randomization lists [67], which are created for each stratum using the software R [68] and the additional package “block edge” [69]. The block length is varied based on random block sequences by the software to avoid predictability of allocation sequences by study personnel.

### Concealment mechanism {16b}

Based on the randomization lists, cards are printed out so that study personnel can assign participants to both intervention groups (tracker-based device alone and tracker-based device in combination with behavioral intervention) and the control group based on sealed envelopes without knowing the allocation in advance.

### Implementation {16c}

The person who creates the randomization lists and cards is not involved in the assignment of participants to the groups and the distribution of the tracker-based devices. Furthermore, the allocation is applied by study personnel who are not involved in the management and analysis of the generated data to keep the persons responsible for the data management and statistics blind to the allocation. The randomization list is locked away by external university staff not involved in the trial for the whole duration of the study.

## Assignment of interventions: Blinding

### Who will be blinded {17a}

Blinding of subjects and study personnel is not possible due to the lack of adequate sham technologies. However, participants do not know the grouping, and outcome assessors are partly blinded with regard to the allocation to the intervention and control groups when analyzing the data from the questionnaires and the diabetes outcomes. Nevertheless, because the tracker-based device generates continuous physical activity data for the intervention group, no blinding is possible when analyzing this data.

### Procedure for unblinding if needed {17b}

Since the risks related to the intervention are regarded as low, emergency unblinding procedures are not necessary.

## Data collection and management

### Plans for assessment and collection of outcomes {18a}

The planning, implementation, outcome assessment, and publication of the study are based on the guidelines and recommendations for ensuring good epidemiological practice [70]. All work steps are recorded in the form of standard operating procedures (SOPs). The procedure for recruiting the participants and procedures for their supervision are recorded in a survey manual. The specialized staff employed for the recruitment and supervision of volunteers is comprehensively trained at the beginning of the study and, if necessary, retrained.

Qualified study personnel will be trained in standardized and reproducible measurement of height, weight, blood pressure, HbA_1c_, and TIR. Furthermore, study personnel are instructed to provide the interventions (tracker-based device alone and tracker-based device in combination with digital coaching) and counseling for adherence in a uniform and reproducible manner.

Socio-demographic and socio-economic factors are measured with standardized indicators of the German Federal Statistical Office [51, 71, 72]. The health-related questions are based on the questionnaire used in the Robert Koch Institute’s German Health Update (GEDA) survey, which includes indicators of the European Health Interview Survey [52]. Therefore, data based on these indicators are comparable with the results of other national and international surveys [73]. Physical activity is measured with the tracker-based device in the intervention group and the Questionnaire for Motor Skills (FFB-mot, [53]) as well as the Movement and Sport Activity Questionnaire (BSA-F, [54]) in the intervention and control group to enable comparison of the different measurements. Both FFB-mot and BSA-F have acceptable psychometric properties [53, 54]. The psychosocial questions are based on questionnaires assessing sport and exercise-related self-efficacy [55, 56], sport and exercise-related volition (Volition in Exercise Questionnaire - VEQ, [57]), behavior change motivation [58], motives and goals with regard to sport and exercise (Bernese Motive and Goal Inventory - BMZI, [59]), affect towards sport and exercise as adapted by Brand [60] from Crites et al. [74], barriers for physical activity as assessed in Brehm et al. [61], and the intention to be physically active [62], as well as the Big Five Inventory (BFI-10, [63]) to assess the subjects’ personality and the Short Scale for Measuring General Self-efficacy Beliefs (ASKU, [64]) to assess the subjects’ general self-efficacy. User satisfaction as well as usability related to the tracker-based device and app are measured with a translated and modified version of the patient version of the mHealth App Usability Questionnaire (MAUQ), which has been shown to be valid and reliable (Cronbach alpha=0.914) [65]. The wearable device (Bangle.js) is also rated by the subjects with a translated version of the Comfort Rating Scale (CRS) by Knight and Baber [75].

The questionnaires are administered on an internet platform for practicability and accuracy reasons.

### Plans to promote participant retention and complete follow-up {18b}

Reminders for the scheduled data assessment appointments and the link to the follow-up online questionnaires will be sent to the participants via e-mail. If participants do not respond or do not have an e-mail account, they are contacted by phone and can fill in the questionnaire at the diabetes center. In addition, to promote participant adherence, the assessments of the medical data take place along with the regular medical visits of the diabetes patients and at times that are comfortable for all participants. To check for selection-related biases, the reasons for non-participation or drop-out and certain characteristics of these participants, such as physical activity behavior and socio-demographic factors, are assessed.

### Data management {19}

To promote data quality, all data will be entered electronically, primarily with closed-ended entry fields. Standardized data entry and coding practices for non-numeric data are described and applied according to the written SOPs. Plausibility tests of the data entries are performed when the data are stored in the databases in a regular manner. In addition, the participant ID used to pseudonymize the participant data and link different data sources has to be entered twice and in a field with a fixed length to minimize errors.

Data are transmitted securely between the tracker-based device, smartphone, databases, and personal computers of the researcher by using appropriate encodings. Furthermore, data are stored in secure databases, which are backed up regularly according to a specific data security plan handled by the University Computing Centre. The databases and personal computers of the researcher are protected with adequate passwords that are changed on a routine basis to prevent unauthorized access. Detailed and further information about data management and data security issues can be found in the data security concept that has been developed for this trial.

### Confidentiality {27}

Data security and confidentiality are guaranteed according to the Regulation (EU) 2016/679 (General Data Protection Regulation) and the Data Protection Act of Lower Saxony. A data security concept has been developed together with the data security officer and data manager at Vechta University. Data collection is limited to data needed to answer the research questions or to safeguard the participants’ rights. All data collection forms and the data processed by the tracker-based device and the app will be identifiable only by the participant ID (an unrelated sequence of characters) to maintain participant confidentiality. All records that contain names or further personal information, such as administrative forms and informed consent forms, will be stored separately from study records and locked away. The databases with the study records will be secured with encrypted connections and password-protected access systems. Study staff will be required to sign agreements to preserve the confidentiality of all participants and are trained in the confidential handling of the data. Only aggregated and anonymized data are made accessible to the public after the end of the study. All other data are deleted after the end of the trial in compliance with data privacy regulations.

### Plans for collection, laboratory evaluation, and storage of biological specimens for genetic or molecular analysis in this trial/future use {33}

No biological specimens will be collected for genetic or molecular analysis in this trial.

## Statistical methods

### Statistical methods for primary and secondary outcomes {20a}

Visual and descriptive (pre-)analyses will be undertaken. All statistical data analyses will be performed with the statistical software R [68]. The primary outcomes (moderate and vigorous physical activity in minutes and steps per week) will be analyzed regarding changes in the percentage of participants that meet the WHO recommendations to be physically active. Therefore, descriptive and explorative time-series analyses will be performed [76]. The overall physical activity as well as the amount of physical activity for different groups will be compared between measurements with the activity tracker and the questionnaire (the BSA-F [54] module) both in minutes per week at t_2_ (post-treatment) using the Bland-Altman method [77].

Since comparisons between participants in the intervention and control groups as well as comparisons of participants at different time points take place, a linear mixed model with two levels will be calculated to test the primary hypotheses [78]. The dependent variable is moderate and vigorous physical activity per week measured with a questionnaire. In the linear mixed model, time (*t*_0_ to *t*_3_) is the intra-class factor, and grouping (intervention group with tracker-based device, intervention group with tracker-based device and digital behavior intervention, and control group) is the inter-class factor [78]. The covariates psychosocial factors, socio-demographic factors, physical and mental health, comorbidities, alcohol and tobacco consumption, nutritional awareness, usage behavior and user satisfaction (with regard to the activity tracker and app), diabetes disease and treatment, participation in external interventions, and severity of the disease are assessed as categorical variables. Random intercepts are assumed while all other effects are regarded as fixed. The null model will be calculated and compared with alternative models regarding its fit via Akaike and Bayesian information criterion [78, 79]. Further, interactions will be considered and will be included in the model if the explanatory power of the model is strengthened. The same analysis will be performed for the assessment of secondary endpoints. Effects of all analyses will be considered significant if *p* < 0.05. All assumptions of the statistical tests will be checked in advance graphically and by appropriate tests. Bonferroni correction will be applied to counteract the problem of multiple testing [80].

### Interim analyses {21b}

Because no potential harm is expected to result from this trial, no interim analyses to terminate the trial are planned.

### Methods for additional analyses (e.g., subgroup analyses) {20b}

Subgroup analyses are planned to be performed by comparing the primary outcomes among healthy individuals, patients with pre-diabetes, and patients with T2DM. Whether the effects of the intervention differ between these subgroups will be tested. Therefore, the health status of the subjects is included as a dummy-variable in the statistical model.

### Methods in analysis to handle protocol non-adherence and any statistical methods to handle missing data {20c}

The collected data will be analyzed in terms of the intention-to-treat (ITT) method, which means that data from all subjects will be assessed because they more realistically reflect the everyday life of the participants [81]. Following the ITT evaluations, the analyses will be carried out again with the per-protocol collective and compared with the results according to ITT using sensitivity analyses [82]. The reasons for withdrawal and drop-out from the trial will be assessed for all groups, and the characteristics of these subjects will be compared between the intervention and control groups. Data will be included in the analyses up to the point of drop-out. The physical activity data measured with activity tracker will be preprocessed using the R package “accelerometry” [83]. Missing and unreliable values will be replaced by the average of neighboring data [83]. For all data, missing values will be quantified and checked if they are not missing at random.

### Plans to give access to the full protocol, participant level-data and statistical code {31c}

At the end of the trial, the aggregated and anonymized data set of the trial will be stored in an appropriate data archive and made available as a scientific use file to other researchers on request to answer similar questions in the field of health research. The data will be available immediately after the end of the study for at least 10 years.

## Oversight and monitoring

### Composition of the coordinating center and trial steering committee {5d}

Not applicable, because no formal committees are needed because of the minimal risks associated with participating in the trial.

### Composition of the data monitoring committee, its role and reporting structure {21a}

No data monitoring committee is needed because of the minimal risks associated with participating in the trial.

### Adverse event reporting and harms {22}

Because of the regular visits to the center in line with routine treatment of the diabetes patients and the regular assessments at four time points for all participants, possible harms associated with the study can be monitored regularly. Furthermore, possible harms and unintended effects of the trial will be reported and assessed by the medical personnel involved in this study. However, the risk of the occurrence of possible harms associated with the trial is regarded as low.

### Frequency and plans for auditing trial conduct {23}

No auditing is planned because the occurrence of possible harms associated with the trial is regarded as low.

### Plans for communicating important protocol amendments to relevant parties (e.g., trial participants, ethical committees) {25}

For any modifications to the protocol that affect the scientific or ethical rigor of the study or the health and safety of the participants, the approval of the Ethics Committee will be obtained before implementation. Furthermore, any changes made to the protocol will be disclosed and published in a formal amendment to the protocol.

### Dissemination plans {31a}

The results of this trial will be made available to interested persons and presented at scientific conferences. All factors that may have influenced the results and their interpretation will be disclosed. All relevant (as well as negative and statistically non-significant) study results will be published in scientific journals and made available to the public on request. The reporting of the results will be based on the CONSORT-EHEALTH (Consolidated Standards of Reporting Trials of Electronic and Mobile EHealth Applications and onLine TeleHealth) guideline for the standardized reporting of the results of trials of internet-based and mobile health interventions [84].

## Discussion

Activity tracker-based consumer electronics are regarded as useful for sustainable improvement of physical activity and for monitoring physical activity in everyday life [20–24, 26, 28]. In addition, with the help of these devices, patients’ adherence to physical activity recommendations can be strengthened [21, 47]. Hence, interventions based on activity trackers have the potential to strengthen physical activity and thus prevent the onset or progression of chronic diseases such as T2DM.

Nevertheless, valid and objectively measured data are needed to understand the complex factors affecting physical activity in daily routine and to increase knowledge about the accuracy as well as reliability of the measurements with these devices [19]. To overcome the limitations of previous studies, such as small sample sizes, short study durations, and lack of analyzing standardized outcomes [20, 26–28, 33–36], the physical activity of 1642 individuals with and without T2DM, half of them wearing the tracker-based device for 6 months, will be assessed and evaluated over 9 months in a randomized, controlled trial. Socio-demographic, socio-economic, physical and mental health-related, and psychological factors that affect physical activity behavior in real-life will be assessed and controlled for in this study. In addition, user behavior related to the tracker and the app and their usability will be studied to evaluate the effects on intervention adherence. Furthermore, the study includes healthy individuals and individuals with pre-diabetes and T2DM to appraise the effects of the intervention against the background of T2DM onset and disease progression. The study uses an interdisciplinary approach, with a team of sports psychologists, sports scientists, health scientists, health care professionals, physicians, and computer scientists, and it involves the processing and analysis of a large amount of data collected with the help of the activity tracker. These factors represent particular strengths as well as challenges in the study.

## Trial status

This is protocol version number 7 14 June 2022. The recruitment is delayed to minimize the risk of a SARS-CoV-2 infection and started in January 2022; the last patient will be enrolled in December 2022. The first results of this study are expected in 2023.

## Data Availability

At the end of the trial, the aggregated and anonymized data set of the trial will be stored in an appropriate data archive and made available as a scientific use file to other researchers on request in order to answer similar questions in the field of health research. The data will be available immediately after the end of the study for at least 10 years.

## Abbreviations

ASKU: Short Scale for Measuring General Self-efficacy Beliefs
BCT: Behavior change techniques
BFI-10: Big Five Inventory
BMZI: Bernese Motive and Goal Inventory
BMI: Body mass index
BSA-F: Movement and Sport Activity Questionnaire
CONSORT: Consolidated Standards of Reporting Trials
CRS: Comfort Rating Scale
FBG: Fasting blood glucose
FFB-mot: Questionnaire for Motor Skills
GEDA: German Health Update
HbA_1c_: Average blood sugar concentrations
ITT: Intention-to-treat
MAUQ: mHealth App Usability Questionnaire
MD: Mean difference
RCT: Randomized controlled trial
(R)CT: Randomized controlled trial or controlled trial
SMD: Standardized mean difference
SOP: Standard operating procedure
TIR: Time in range
VEQ: Volition in Exercise Questionnaire
WHO: World Health Organization
